# Lacking Warmth and Competence: How Younger Adults Utilize “OK Boomer” According to the Stereotype Content Model

**DOI:** 10.1093/geroni/igab046.2313

**Published:** 2021-12-17

**Authors:** Katelyn Frey, Michael Vale, Toni Bisconti

**Affiliations:** 1 University of Akron, Akron, Ohio, United States; 2 Sacred Heart University , Trumbull, Connecticut, United States; 3 The University of Akron, Akron, Ohio, United States

## Abstract

Younger adults have coined the popular retort “OK Boomer,” referring to the 76 million Baby Boomers born between 1946-1964. The Stereotype Content Model (SCM) is a framework used to assess stereotypical perceptions of various groups, and older adults generally fall in the paternalistic “high warmth/low competence” quadrant. The stereotypes behind “OK Boomer” could correspond to any of the four quadrants of the SCM. The present study's goals were to determine the parameters for using the phrase, how hostile and benevolent ageism may underlie its use, and whether or not the eponymous “Boomer” fits into the same cluster in the SCM as older adults in general. In a sample of 316 participants (18-33; M = 23; SD = 5.25), we found that age was related to using “OK Boomer” such that being younger is associated with feeling more comfortable using the phrase in front of anyone (r = -.208, p < .01), using the phrase more frequently (r = -.218, p < .01), and sharing “OK Boomer” memes, pictures, and jokes online (r = -.203, p < .01). Hostile ageism, but not benevolent, was associated with an increased likelihood of saying “OK Boomer” in front of anyone (r = .256, p < .01), to use it more frequently (r = .242, p < .01), and to share “OK Boomer” jokes online (r = .301, p < .01). Content analysis results indicate that “OK Boomer” does not correspond to the paternalistic quadrant of the SCM due to Boomers’ perceived low warmth.

